# Chronic low‐grade inflammation in heart failure with preserved ejection fraction

**DOI:** 10.1111/acel.13453

**Published:** 2021-08-12

**Authors:** Thassio Mesquita, Yen‐Nien Lin, Ahmed Ibrahim

**Affiliations:** ^1^ Cedars‐Sinai Medical Center Smidt Heart Institute Los Angeles CA USA; ^2^ Division of Cardiovascular Medicine Department of Medicine China Medical University and Hospital Taichung Taiwan

**Keywords:** aging, arrhythmias, diastolic dysfunction, fibrosis, heart failure with preserved ejection fraction, inflammasome, inflammation

## Abstract

Heart failure (HF) with preserved ejection fraction (HFpEF) is currently the predominant form of HF with a dramatic increase in risk with age. Low‐grade inflammation, as occurs with aging (termed “inflammaging”), is a common feature of HFpEF pathology. Suppression of proinflammatory pathways has been associated with attenuated HFpEF disease severity and better outcomes. From this perspective, inflammasome signaling plays a central role in mediating chronic inflammation and cardiovascular disease progression. However, the causal link between the inflammasome‐immune signaling axis on the age‐dependent progression of HFpEF remains conjectural. In this review, we summarize the current understanding of the role of inflammatory pathways in age‐dependent cardiac function decline. We will also evaluate recent advances and evidence regarding the inflammatory pathway in the pathophysiology of HFpEF, with special attention to inflammasome signaling.

## Graphical abstract

Heart failure (HF) with preserved ejection fraction (HFpEF) is a complex syndrome characterized by multisystem disorders. Despite the increasing age‐dependent prevalence, there are very few effective therapies. Considered now as the greatest unmet medical need in cardiovascular disease, comorbidity‐driven inflammation has emerged as a critical component of HFpEF pathogenesis. Thus, chronic low‐grade inflammation may drive key pathophysiological changes in HFpEF.

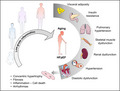

**Abbreviations**
AF: atrial fibrillation
COPD: chronic obstructive disease
HF: heart failure
HFpEF: heart failure with preserved ejection fraction
HFrEF: heart failure with reduced ejection fraction
IL: interleukin
LV: left ventricle
NLRP3: nucleotide‐binding oligomerization domain leucine‐rich repeat and pyrin domain‐containing protein 3

## 1 INTRODUCTION

**FIGURE 1:**
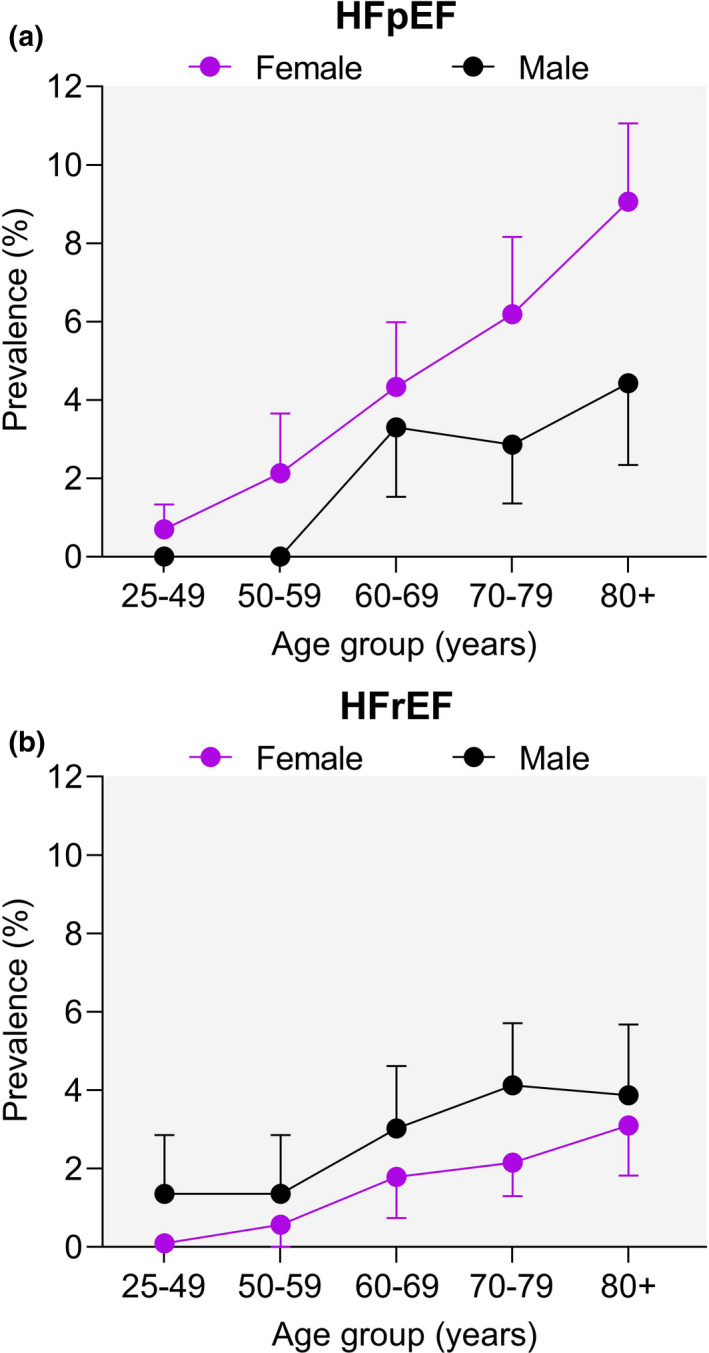
Epidemiological prevalence of HFpEF and HFrEF by age and sex. Increasing prevalence of heart failure with preserved ejection fraction (HFpEF, a) and reduced ejection fraction (HFrEF, b) with age in both sexes. Reproduced with permission from (Ceia et al., 2002). Copyright © 1999–2021, John Wiley & Sons

Heart failure (HF) is a growing public health problem worldwide exerting heavy burdens on productivity, morbidity, mortality, and healthcare expenditure. Heart failure is highly prevalent in elderly people and is associated with multi‐morbid illness. Hypertension, sarcopenia, diabetes, obesity, pulmonary congestion, and atherosclerosis‐related vascular diseases are common independent risk factors for developing HF (Wagner & Dimmeler, 2020). Although the lifetime risk of developing HF for both men and women at age 80 is ~20%, community‐based studies revealed differential gender‐associated burden in patients with HF with preserved ejection fraction (HFpEF) compared to HF with reduced ejection fraction (HFrEF; Ceia et al., 2002; Dunlay et al., 2017; Eisenberg et al., 2018). Figure 1 shows that the incidence of HF progressively increases for both types of HF in an age‐dependent manner, but the prevalence of HFpEF at any given age was higher in women compared to men (Ceia et al., 2002; Dunlay et al., 2017) Moreover, the severity of HFpEF increases more rapidly with age than HFrEF, suggesting a close interplay between aging and age‐related HFpEF progression compared to HFrEF.

The absence of effective treatments is perhaps explained by the heterogeneity of HFpEF syndrome and the traditional assumption that the therapeutic interventions for HFrEF will also be effective for HFpEF (Shah et al., 2016). Evolving understanding points to divergent etiologies of HFpEF versus HFrEF. For instance, HFrEF is driven largely by inflammation‐induced damage and volume overload. This inflammation can occur in a sterile context including coronary artery disease, tissue necrosis from post‐ischemic damage, toxic exposure, acute physical trauma, hemorrhage, or resuscitation. Infections including viral myocarditis also cause inflammation that contributes to HFrEF (Simmonds et al., 2020). While inflammation also plays a major role in HFpEF, the etiology of HFpEF is more profuse and includes metabolic syndrome, hypertension, pulmonary, and liver disease. Elevated circulating inflammatory biomarkers such as C‐reactive protein (CRP), interleukin‐1 (IL‐1), and tumor necrosis factor‐alpha (TNFα) are more pronounced in HFpEF than in HFrEF (DuBrock et al., 2018; Kalogeropoulos et al., 2010; Sanders‐van Wijk et al., 2015). This substantiates the comorbidity‐inflammation paradigm for HFpEF (Paulus & Zile, 2021). These divergent etiologies result in different hemodynamic and metabolic load‐induced proinflammatory signaling, which manifest distinct forms of HF phenotypes (Paulus & Zile, 2021). More detailed comparisons between HFpEF and HFrEF are found elsewhere (DeBerge et al., 2019; Paulus & Dal Canto, 2018; Simmonds et al., 2020). Therefore, effective treatments for HFpEF must tailor to the pathophysiological pathways that uniquely drive this disease. This necessitates a more comprehensive molecular and cellular understanding of HFpEF syndrome. To this end, considerable attention has been given to the unique inflammatory signaling in HFpEF (Edelmann et al., 2015; Kalogeropoulos et al., 2010; Matsubara et al., 2011).

Activation of proinflammatory pathways is a remarkable feature in HFpEF patients (Edelmann et al., 2015; Kalogeropoulos et al., 2010; Matsubara et al., 2011), which also coincides with aging‐induced inflammation (termed “inflammaging”). Furthermore, there is substantial evidence that inflammaging plays a causative role in HFpEF. In this review, we summarize the current knowledge of inflammatory mechanisms driving the age‐dependent decline in cardiac function. We focus on HFpEF‐relevant inflammatory mechanisms, with special attention to the inflammasome signaling. We also evaluate recent evidence regarding the inflammatory pathway in the pathophysiology of HFpEF.

## 2 THE NLRP3 INFLAMMASOME: A BRIEF OVERVIEW

Originally described in 2002 (Martinon et al., 2002), inflammasomes are important signaling pathways in somatic cells in response to sterile or non‐sterile insult. Inflammasome signaling complexes comprise three main components: sensor molecules, adaptor protein apoptosis‐associated speck‐like proteins containing a caspase recruitment domain (ASC), and caspase‐1 (Lee & Kang, 2019). Four classes of inflammasome signaling mediators have been described, including nucleotide‐binding oligomerization domain leucine‐rich repeat and pyrin domain‐containing protein 3 (NLRP3), NLRP1, absent in melanoma 2 (AIM2), and NLR family caspase activation and recruitment domain‐containing protein 4 (NLRC4; Christgen et al., 2020; Lamkanfi & Dixit, 2014; Yang et al., 2019). However, NLRP3 is the best‐characterized inflammasomes sensor in the heart (Swanson et al., 2019; Yao et al., 2018).

### 2.1 Canonical inflammasome priming and activation

Inflammasome signaling is tightly regulated through distinct canonical and non‐canonical pathways. Both pathways, however, result in cell death and the release of proinflammatory cytokines. Canonical NLRP3 inflammasome signaling is mediated through two steps: priming followed by activation (Swanson et al., 2019). Priming is mediated by activation of pattern recognition receptors (PRRs) including toll‐like receptors (TLR), intracellular sensors like nucleotide‐binding oligomerization domain‐containing protein 2 (NOD2), or cytokines receptors. Activation of these receptors leads to activation of nuclear factor‐κB (NF‐κB), and transcriptional upregulation of NLRP3, interleukins (ILs), and caspase‐1 (Bauernfeind et al., 2009; Xing et al., 2017). Multiple NLRP3 post‐translational modifications have been identified as regulators of priming signals including ubiquitination, phosphorylation, and sumoylation (Barry et al., 2018; Juliana et al., 2012; Liu et al., 2016; Song et al., 2017). While NF‐κB activation is necessary for inflammasome signaling, it is not sufficient for inflammasome complex assembly (Bauernfeind et al., 2009; Zhou et al., 2011).

Following the activation of the NLRP3 cascade, ASC recruits pro‐caspase‐1 through the N‐terminal caspase recruitment (CARD) domain to assemble the NLRP3 inflammasome. Subsequently, the active form of caspase‐1 cleaves pro‐IL‐1β and pro‐IL‐18 to their active proinflammatory forms (Lamkanfi & Dixit, 2012; Lee & Kang, 2019). Moreover, several stimuli have been reported as facilitators of NLRP3 activation assembly, including mitochondrial dysfunction, reactive oxygen species (ROS), lysosomal disruption, extracellular osmolarity, or pH alterations, degradation products of extracellular matrix components, potassium efflux, and increased of cytosolic calcium (Swanson et al., 2019; Zhou et al., 2011). Active caspase‐1 also promotes pyroptosis, which may cause cell permeability by forming membrane pores and release of proinflammatory, such as IL‐1β and IL‐18 (Liu et al., 2016). Pyroptosis is triggered by the cleavage of gasdermin D (GSDMD) by activated caspase‐1 (Shi et al., 2015), which, in turn, exacerbates the inflammation and triggers cell necrosis (Liu et al., 2016; Shi et al., 2015).

### 2.2 Non‐canonical inflammasome priming and activation

**FIGURE 2:**
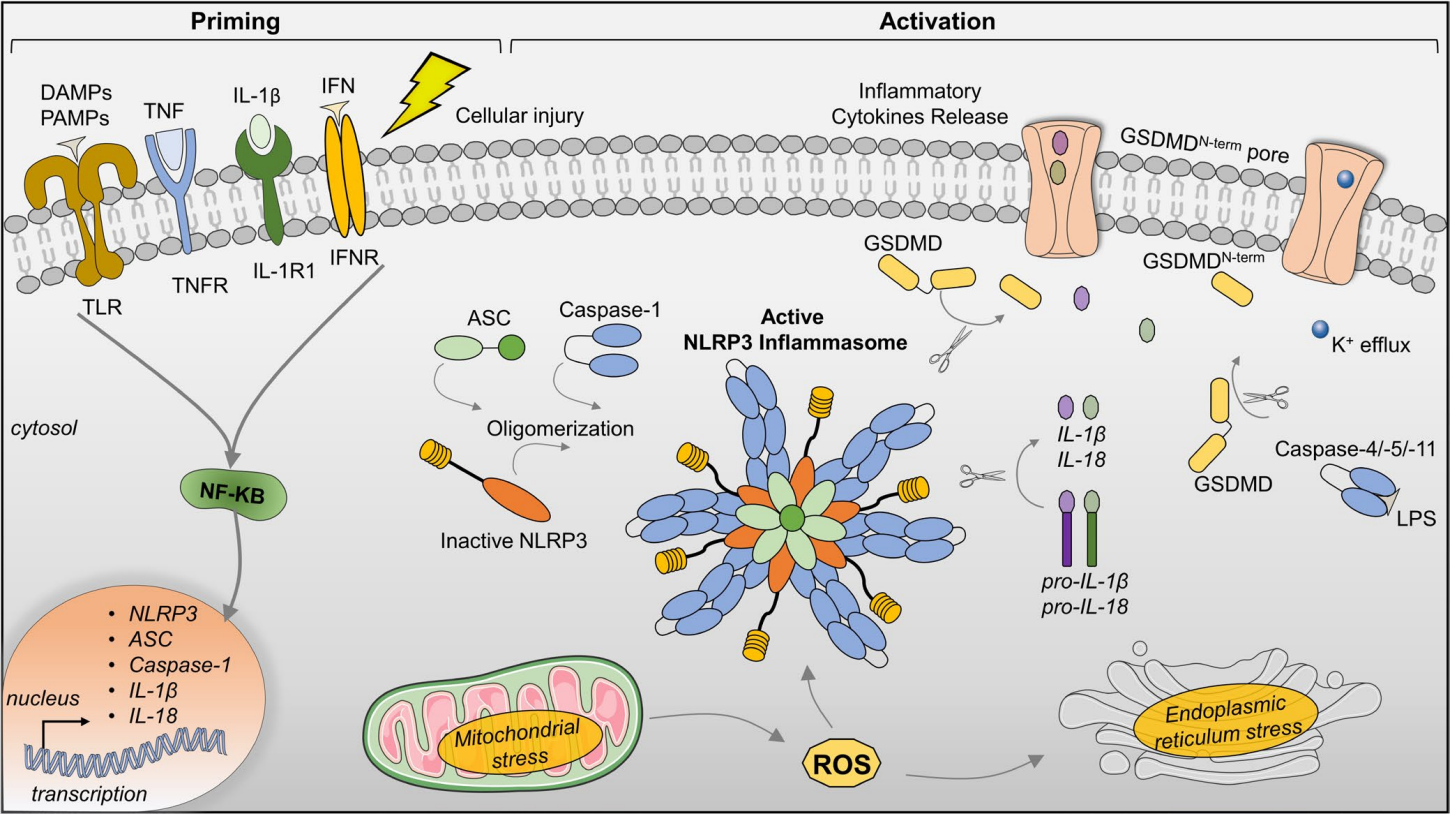
NLRP3 inflammasome priming and activation. The priming signaling occurs by the activation of cytokines, damage‐associated molecular patterns (DAMPs), or pathogen‐associated molecular patterns (PAMPs), leading to the transcriptional activity of nuclear factor (NF)‐κB and upregulation of inflammasome components and cytokines (IL‐1β and IL‐18). Priming signals activate a complex cascade of events including potassium (K^+^) efflux, endoplasmic reticulum, and mitochondrial stress (Activation signaling). Activation of the NLRP3 inflammasome complex involves NLRP3 oligomerization, ASC polymerization, and recruitment of pro‐caspase‐1. Active NLRP3 inflammasome complex and pro‐caspase‐1 cleave pro‐IL‐1β and pro‐IL‐18 into the active forms, IL‐1β and pro‐IL‐18. Gasdermin D (GSDMD) is also cleaved (GSDMD^Nterm^) forming pores and inducing pyroptosis. Cleavage of GSDMD also occurs via the non‐canonical pyroptosis pathway upon activation of caspases‐4/‐5/‐11 by cytosolic lipopolysaccharide (LPS). ASC, apoptosis‐associated speck‐like protein containing a CARD; GSDMD^Nterm^, GSDMD amino‐terminal cell death domain; IFN, interferon; IFNR, interferon α/β receptor; IL‐1R1, interleukin‐1 receptor type 1; IL‐1β, interleukin 1β; NLRP3, NACHT, LRR, and PYD domains‐containing protein 3; ROS, reactive oxygen species; TLR, Toll‐like receptor; TNF, tumor necrosis factor; TNFR, tumor necrosis factor receptor. This figure was created in part with modified Servier Medical Art templates

Non‐canonical inflammasome activation occurs by direct recognition of intracellular lipopolysaccharides (LPS) or other pathogens by caspases‐1, 4, and ‐5 (in humans) and caspase‐1 and ‐11 in mice (Hagar et al., 2013; Kayagaki et al., 2013). Intracellular LPS causes caspase‐11 oligomerization and activation by autoproteolytic cleavage (Lee et al., 2018). Active caspase‐1 can also enhance canonical caspase‐1 function in activating IL‐1β and IL‐18. This convergence suggests the interdependence of canonical and non‐canonical inflammasome signaling (Rühl & Broz, 2015; Schmid‐Burgk et al., 2015). Like the canonical pathway, the activation of caspase‐4,‐5,‐1 leads to the cleavage of GSDMD and consequent pyroptosis activation (Baker et al., 2015; Kayagaki et al., 2015). Although non‐canonical inflammasome can trigger pyroptosis, it does not directly process proinflammatory IL‐1β and IL‐18. Exceptions to this two‐step model of NLRP3 inflammasome activation (Latz et al., 2013) have been observed in other model systems which may point to differences between human and murine signaling. For instance, human cells are more sensitive to NLRP3 inflammasome activation compared to murine tissue (Wang et al., 2013). Figure 2 illustrates canonical and non‐canonical NLRP3 inflammasome priming and activation steps.

### 2.3 NLRP3 inflammasome signaling in the heart

Although canonical NLRP3 inflammasome signaling in the innate immune cells is well‐described, its role in non‐immune cells including cardiac cells is less understood. The expression of NLRP3 inflammasome components has been identified in cardiomyocytes and cardiac fibroblasts, which are the two most abundant cell types in the heart (Litviňuková et al., 2020). Despite the low abundance of NLRP3 inflammasome components in the healthy heart, the expression markedly increases in cardiomyocytes, cardiac fibroblasts, leukocytes, and endothelial cells following injury (Mezzaroma et al., 2011; Yin et al., 2009).

Several studies have demonstrated the central role of NLRP3 inflammasome in cardiovascular diseases (Bracey et al., 2013; Liu et al., 2014; Mezzaroma et al., 2011; Toldo et al., 2016; Valle Raleigh et al., 2017). Upregulation of NLRP3 inflammasome signaling occurs after myocardial infarction (MI), ischemic heart disease, hypertension, diabetic cardiomyopathy, and chronic HF (Bullón et al., 2017; Liu et al., 2018; Van Tassell et al., 2013). Of note, inhibition of NLRP3 with small interfering RNA prevented inflammasome activation and cardiac cell death, thereby ameliorating the extent of damage and subsequent maladaptive myocardial remodeling after MI (Mezzaroma et al., 2011).

Inflammatory cells, especially macrophages, produce a large amount of IL‐1β in response to NLRP3 inflammasome activation. Cardiac fibroblasts are also responsible for the NLRP3 inflammasome‐driven IL‐1β production in MI (Sandanger et al., 2013). However, activation of NLRP3 inflammasome in cardiomyocytes leads to caspase‐1‐dependent cell death, pyroptosis, but not IL‐1β release (Mezzaroma et al., 2011). Together, these studies suggest that activation NLRP3 inflammasome drives IL‐1β release in cardiac fibroblasts and death in cardiomyocytes, indicating a cell‐specific inflammatory response.

The role of NLRP3 Inflammasome was also demonstrated in cardiomyocytes from patients with paroxysmal and long‐standing persistent (chronic) atrial fibrillation (AF; Yao et al., 2018). Interestingly, distinct NLRP3 inflammasome activity was observed in these types of AF. Atrial myocytes from patients with paroxysmal AF have higher NLRP3 inflammasome activity due to an increased activation step, while the priming processes remained unchanged. On the other hand, both steps (priming and activation) were upregulated in patients with chronic AF (Yao et al., 2018). This study was among the first to demonstrate a link between elevated inflammasome activity in atrial myocytes and the structural remodeling, electrophysiological changes, and calcium mishandling features seen in AF.

## 3 CARDIAC INFLAMMAGING

Aging is a risk factor for cardiovascular diseases as the prevalence increases from 40% in people 40–59 years old to 75% in people 60–79 years and 86% for those above (Rodgers et al., 2019). Cardiac aging is accompanied by molecular, cellular, and structural changes that lead to functional decline and, eventually, HF. Accelerated cardiomyocyte senescence is driven by telomere‐shortening (Blasco, 2007), DNA damage (Edgar et al., 2009), oxidative stress (Hekimi et al., 2011), inflammatory recruitment (Kalogeropoulos et al., 2010), and other cytotoxic pathways (Kajstura et al., 2000; Papp et al., 2012; Sheydina et al., 2011). Senescent cells also perpetuate tissue senescence by releasing paracrine factors, including proinflammatory cytokines, chemokines, growth factors, and proteases (Coppé et al., 2010; Jeyapalan & Sedivy, 2008). For instance, inflammatory cytokines (IL‐1, IL‐6, IL‐8), matrix metalloproteases, and fibroblast‐derived collagens (type 1a) perpetuate stress in neighboring cardiomyocytes (Postmus et al., 2019; Siddiqi & Sussman, 2013). Chronic inflammation during aging negatively impacts cell contractility, metabolism, intercellular communication, and extracellular components (Feridooni et al., 2015; Hulsmans et al., 2018). Thus, the accumulation of senescent cells with age contributes to inflammation, fibrosis, and further cardiomyocyte loss. This forward feedback mechanism ultimately leads to impaired cardiac function (Campisi, 2011; Hall et al., 2016).

In the heart, most of cell types can turn senescent in a cell type‐specific manner. For instance, single‐nucleus sequencing from aging cardiac tissue revealed higher expression of plasminogen activator inhibitor‐1 (PAI‐1), which may impair endothelial angiogenesis and cardiac vascularization (Vidal et al., 2019). In aged hearts, the endothelial expression of laminin shifts from β2 to β1 form contributing to cell‐matrix adhesion and modulating of endothelial‐to‐mesenchymal transition, which compromises arterial integrity (Wagner et al., 2018). Senescing cardiac progenitors upregulate senescent markers (SA‐β‐gal, p16Ink4a, and γH2AX) which impair their ability to repair cardiac tissue after damage (Lewis‐McDougall et al., 2019). Also, the secretome of mesenchymal stromal cells from aging hearts contributes to the recruitment of C‐C chemokine receptor type 2 (CCR2)^+^‐ monocytes, which secrete IL‐1β (Martini et al., 2019). Though the early inflammatory response is important for the clearance of dead cells and facilitates reparative processes, chronic inflammation contributes to long‐term pathophysiological remodeling of the heart (Frangogiannis, 2014).

Impaired left ventricular (LV) diastolic function is a hallmark of cardiac aging. Age‐related diastolic dysfunction features prominently in HFpEF, through impaired relaxation, myocardial and myocyte stiffening, and associated changes in filling dynamics (Sharma & Kass, 2014). Although the systolic function remains preserved during aging (Feridooni et al., 2015; Lakatta & Levy, 2003), reduced cardiac reserve and chronotropic response during exercise substantiate an age‐associated decline of function. The thickening of the LV has also been observed (Liu et al., 2013) in response to cardiomyocyte senescence (Olivetti et al., 1991). Part of compensatory aging‐related LV remodeling also involves changes in the extracellular matrix composition. Increased age‐related interstitial LV fibrosis contributes to the loss of elastic properties and impaired electrical conduction, which can eventually lead to diastolic dysfunction and arrhythmias (Biernacka & Frangogiannis, 2011; Curtis et al., 2018; Gazoti Debessa et al., 2001; Liu et al., 2013; Wang & Shah, 2015). Aged hearts also show fibrotic remodeling in the cardiac conduction system (sinoatrial and atrioventricular nodes, the bundle of His, bundle branches, and Purkinje fibers), which favors the development of conduction blocks (Song et al., 1999). Other aging‐associated mechanisms that contribute to the decline of cardiac function are extensively described elsewhere (Ferrucci & Fabbri, 2018; Gude et al., 2018; Wagner & Dimmeler, 2020).

### 3.1 Cardiac NLRP3 inflammasome during aging

Age‐related systemic inflammation underlies several chronic degenerative disorders that drive mortality in the elderly population (Ferrucci & Fabbri, 2018; Wagner & Dimmeler, 2020). Nucleotide‐binding oligomerization domain leucine‐rich repeat and pyrin domain‐containing protein 3 inflammasome signaling is critical for geriatric inflammation. Nucleotide‐binding oligomerization domain leucine‐rich repeat and pyrin domain‐containing protein 3 deficiency exerts a protective effect on systemic inflammation (Grant & Dixit, 2013; Vandanmagsar et al., 2011; Wen et al., 2011). The predominant role of IL‐1β was already demonstrated in several age‐related degenerative diseases including type 2 diabetes and Alzheimer's disease (Heneka et al., 2013; Youm et al., 2011). Given aging‐associated systemic inflammation and the fact that inflammasome players are expressed in several organs, there is compelling evidence demonstrating that age‐related activation of NLRP3 inflammasome signaling causes a functional decline in multiple organs (Marín‐Aguilar et al., 2020; Youm et al., 2013). Mice with specific deletion of inflammasome components revealed attenuated functional decline with aging and enhanced longevity (Marín‐Aguilar et al., 2020; Youm et al., 2013). Moreover, the enhanced lifespan observed in these mice was coupled with cardiac preservation, attenuated myocyte hypertrophy, cardiac fibrosis, and preserved cardiac function (Marín‐Aguilar et al., 2020). Although it remains conjectural, it has been suggested that the observed longevity could be related to the regulation of NLRP3 inflammasome by AMP‐activated protein kinase (AMPK; Cordero et al., 2018). Together, these findings suggest that the NLRP3 inflammasome contributes to the inflammaging process.

Besides aging, hypertension, and obesity are the two dominant comorbidities within the HFpEF population (Dunlay et al., 2017). Metabolic dysfunction is a feature of aging; a large proportion of HFpEF individuals are overweight or obese (Kitzman & Lam, 2017; Obokata et al., 2017). Clinical and preclinical studies linked adiposity with deteriorating cardio‐pulmonary parameters in HFpEF (Obokata et al., 2017; Schiattarella et al., 2021). A recent study created a novel HFpEF mouse model that integrated aging, obesity (high‐fat diet), and hypertension (desoxycorticosterone pivalate stimulation; (Deng et al., 2021). Among the prominent features of this model were systemic inflammation as measured overproduction of IL‐1β and IL‐18 and NLPR3 inflammasome activity (Deng et al., 2021). These findings further substantiate the hypothesis that HFpEF is a cardiometabolic‐driven inflammatory disease (Schiattarella et al., 2021). Although the metabolic‐driven inflammation likely occurs before the aging of the population (inflammaging processes), these events synergically act to facilitate HFpEF pathology. Thus, future studies are necessary to determine the interplay between senescence mechanisms and metabolic‐induced inflammation for the HFpEF pathogenesis.

Cardiovascular‐related mortality occurs in ~60% of all deaths in HFpEF patients. Nearly 25% of these mortalities occur by sudden cardiac death (Zile et al., 2010). Significant structural and electrical remodeling of cardiac tissue has been linked with experimental evidence of delayed ventricular repolarization and higher susceptibility to ventricular tachycardia (Cho et al., 2017, 2018), preceding the sudden death in HFpEF animals (Cho et al., 2018). However, AF is the most frequent form of arrhythmia in HFpEF (Reddy et al., 2020). In ZSF1 obese rats, isolated atrial myocytes had increased mitochondrial fission and elevated ROS production indicating a role of cardiometabolic inflammation (Bode et al., 2020). Moreover, calcium mishandling in left atrial cardiomyocytes of HFpEF rats was attenuated by administration of the anti‐inflammatory cytokine, IL‐10 (Bode et al., 2020). Atrial fibrillation has also been observed in a HFpEF model without obesity (Shuai et al., 2020). Uninephrectomy mice with continuous aldosterone and salt diet recapitulated some features of HFpEF, including AF (Shuai et al., 2020). Increased atrial fibrosis and AF inducibility occur along with elevated expression of TNFα, IL‐6, and IL‐1β (Shuai et al., 2020). Activation of inflammatory pathways initiates atrial fibrotic reprogramming (Hu et al., 2015). However, preliminary findings in a two‐hit HFpEF model (Schiattarella et al., 2019) showed no atrial fibrosis despite the increased AF inducibility (Tong Dan et al., 2020). Thus, further investigation to fully describe the role of inflammation in HFpEF‐related AF.

Aging is an independent risk factor for the development of most arrhythmias (Curtis et al., 2018). Age‐associated atrial structural remodeling due to fibrosis leads to a loss of synchrony, and stiffness leads to a loss of compliance. Electrical changes in the atria include heterogeneity in action potential duration and conduction slowing (Curtis et al., 2018; Kistler et al., 2004). Together, these changes create an environment that favors the initiation and perpetuation of AF. Our group recently provided the first evidence that the NLRP3 inflammasome links HFpEF with AF in an age‐ and gender‐dependent HFpEF model (Mesquita et al., 2020). Aged female Fischer 344 rats with HFpEF exhibited higher AF inducibility compared to young control rats or age‐matched rats without HFpEF. Our findings also revealed that atrial activation of NLRP3 inflammasome was not associated with enhanced expression of cleaved IL‐1β and IL‐18. However, increased cleavage of GSDMD indicates that activation of NLRP3 inflammasome in atrium tissue of HFpEF animals is associated with non‐canonical activation of inflammasome signaling (Mesquita et al., 2020).

## 4 HFPEF: A PERMEATING INFLAMMATORY DISEASE

In contrast to the associative role of inflammation in HFrEF, HFpEF is thought to be caused by a combination of systemic and local inflammatory factors (DeBerge et al., 2019; Paulus & Tschöpe, 2013). A rigorous interrogation of HFpEF patient biopsies revealed that concentric remodeling (Zile et al., 2011), cardiomyocyte stiffness (Borbély et al., 2005), hypertrophy (van Heerebeek et al., 2006), and interstitial fibrosis (Kasner et al., 2011) are deeply influenced by the chronic systemic inflammation. This local maladaptive tissue remodeling has been associated with elevated serum proinflammatory cytokines, which are predictive of poor clinical outcomes in HFrEF and HFpEF (Abernethy et al., 2018; Collier et al., 2011; DeBerge et al., 2019). Thus, we discuss findings regarding the role of inflammation and its resolution in the context of HF, with a focus on HFpEF.

### 4.1 The role of systemic inflammation in the development of HFpEF

**FIGURE 3:**
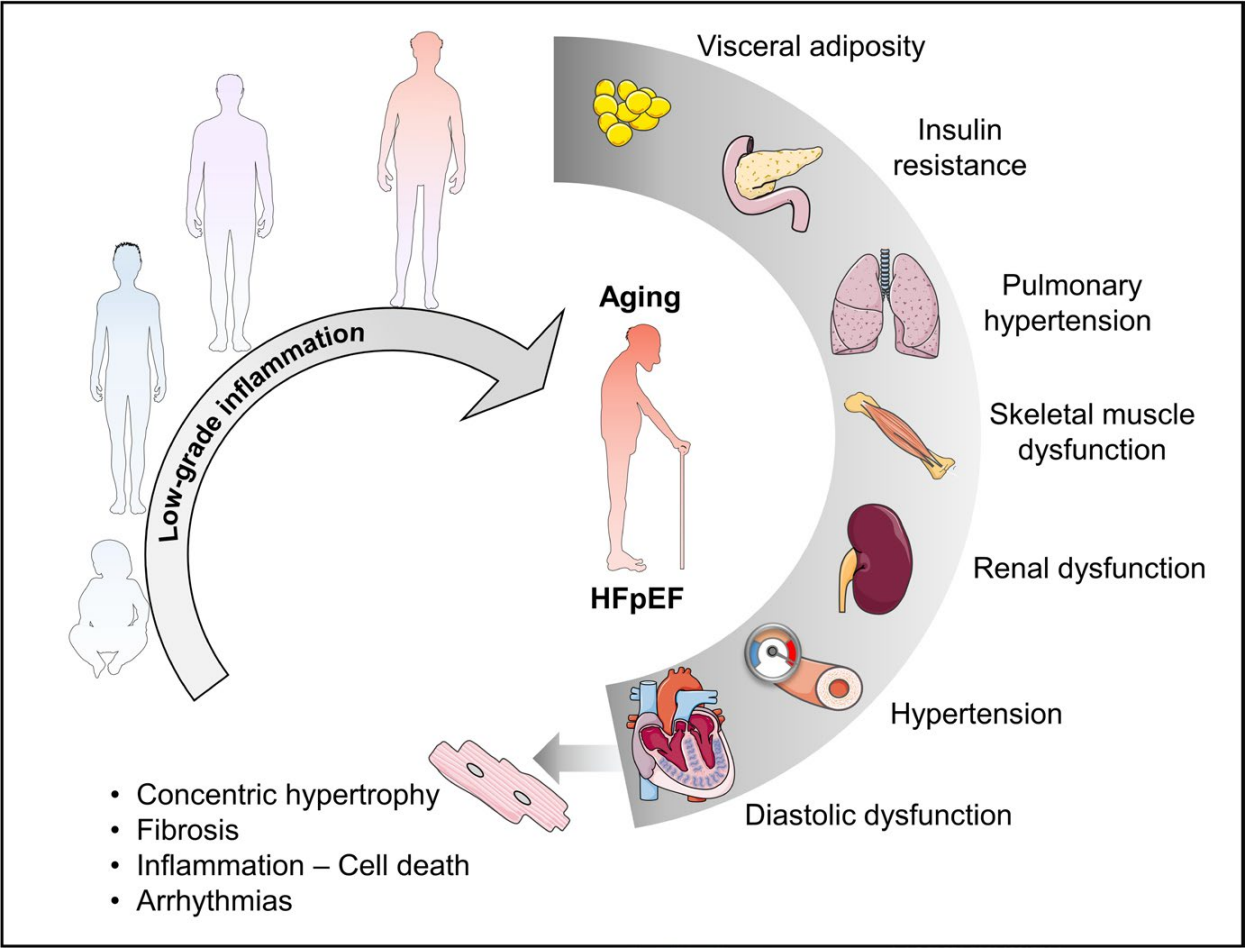
Influential chronic low‐grade inflammasome in the development of HFpEF comorbidities. The collective impact of systemic inflammation caused by multiorgan dysfunction plays an influential role in HFpEF pathogenesis, which is associated with myocardial stiffness, concentric remodeling, cell death, and arrhythmias. This figure was created in part with modified Servier Medical Art templates

Non‐cardiac comorbidities are highly prevalent and usually precede (Lam et al., 2011) the development of HFpEF (Ather et al., 2012). The most highly represented include hypertension (Slivnick & Lampert, 2019), pulmonary hypertension (PH; Rosenkranz et al., 2019), obesity (Savji et al., 2018), diabetes (McHugh et al., 2019), chronic obstructive pulmonary disease (COPD; Streng et al., 2018), chronic kidney disease (CKD; Mavrakanas et al., 2019), and coronary artery disease (Shah et al., 2018). More than 50% of patients with HFpEF present with at least five of these comorbidities (Eisenberg et al., 2018). The salient feature among these diseases is the presence of a systemic inflammatory state. The greatest evidence of systemic inflammatory state in HFpEF patients comes from data of peripheral blood biomarkers, which commonly identify elevated levels of inflammatory biomarkers including CRP, IL‐1β, IL‐6, IL‐10, immunoglobulin‐like transcript 6, TNFα, and myeloperoxidase (MPO; Chirinos et al., 2020; DuBrock et al., 2018; Sanders‐van Wijk et al., 2020). Figure 3 summarizes the inflammatory paradigm of HFpEF pathophysiology. For instance, increased arteriole pressure triggers increased ROS production and release of proinflammatory cytokines in the renal or pulmonary vasculature in hypertensive HFpEF patients (Collier et al., 2011) and animal models (Hummel et al., 2012; Tian et al., 2007). In obesity, inflammation is mediated by the infiltration of monocytes and macrophages in visceral white adipose tissue, which amplifies proinflammatory cytokine release and elevation of these markers in the serum. In type II diabetes, insulin resistance triggers systemic inflammation through glucose‐mediated redox stress and activation of the vascular inflammasome (Sharma et al., 2018). Finally, in COPD and CKD, tissue damage triggers a cascade of leukocyte mobilization which maintains systemic inflammation. The most salient inflammatory markers in the aforementioned conditions include IL‐6, TNFα (van Heerebeek et al., 2006), and monocyte chemoattractant protein (MCP‐1; Abernethy et al., 2018; Collier et al., 2011). Indeed, elevated circulating levels of IL‐6 and TNFα are highly predictive of HFpEF incidence but not of HFrEF, suggesting a more central role of inflammation in the former than the latter (Kalogeropoulos et al., 2010). Subsequent studies identified two additional inflammatory markers that were highly correlated with HFpEF including soluble ST2 (sST2; Shah et al., 2011) and pentraxin 3 (Matsubara et al., 2011). Importantly, elevated systemic levels of inflammatory biomarkers positively correlate with acute decompensation in HFpEF and HF severity (Abernethy et al., 2018). While the presence of circulating inflammatory markers indicates a proinflammatory state, the source of these factors is not entirely known. Incorporation of inflammatory biomarkers as input variables into machine learning‐based clustering of HFpEF subpopulations identified 3 phenogroups (Sabbah et al., 2020). Interestingly, the pan‐inflammatory phenotype with the highest circulating levels of inflammatory mediators (and worst outcome), coincided with elevated myocardial fibrosis biomarkers (Sabbah et al., 2020). This suggests that circulating inflammatory markers originate from damaged myocardial tissue. However, the causal relationship between serum inflammatory markers and inflammasome activity in the heart remains to be investigated.

### 4.2 The transition from systemic inflammation to myocardial inflammation

The presence of sustained systemic inflammation eventually triggers insult in organ tissue. It remains unclear where these transition point(s) occur, but preliminary evidence suggests that one transition point occurs by way of the coronary endothelium. Circulating proinflammatory cytokines trigger the upregulation of vascular cell adhesion molecule‐1 (VCAM1) and E‐selectin (van Heerebeek et al., 2008; Westermann et al., 2011). However, upregulation of NADPH 2, E‐selectin, intercellular cell adhesion molecule‐1 (ICAM1), and VCAM1 was observed in macrophages and endothelial cells but not in cardiomyocytes in the myocardium of HFpEF patients and obese ZSF1‐HFpEF (Franssen et al., 2016). Expression of these homing molecules leads to mobilization and infiltration of leukocytes to endothelial tissue. Proinflammatory cytokines by infiltrating leukocytes exert stress that causes the generation of ROS. This oxidative condition causes mitochondrial fragmentation further amplifying cell death and inflammation, and transdifferentiation of endothelial and fibroblasts into the profibrotic myofibroblasts via endothelial‐to‐mesenchymal transition (EMT; Griendling et al., 2000; Kovacic et al., 2019).

Cardiac nitrosative and oxidative stress have been observed in human and animal HFpEF models (Schiattarella et al., 2019; van Heerebeek et al., 2012; Westermann et al., 2011). Through these pathways, decreased nitric oxide (NO) bioavailability is observed in HFpEF patients, which may explain the impaired vasodilation response to acetylcholine stimulation in the coronary capillary bed (Yang et al., 2020). The mechanistic link between the cardiac remodeling observed in HFpEF with inflammation and impaired NO‐dependent signaling from endothelial cells to cardiomyocytes was demonstrated (Franssen et al., 2016). Reduced NO availability in the coronary endothelium suppresses cyclic guanosine monophosphate (cGMP)‐protein kinase G (PKG) signaling. Nitric oxide is a negative regulator of NLRP3 inflammasome activation (Hernandez‐Cuellar et al., 2012; Mao et al., 2013), therefore, reduced bioavailability of NO in HFpEF may facilitate inflammasome activation. An analysis of HFpEF cardiac homogenates showed reduced levels of PKG and cGMP (van Heerebeek et al., 2012). Protein kinase G is also an inducer of vascular smooth muscle dilation and arteriolar relaxation. PKGI (the PKG splice variant expression in cardiovascular tissue) phosphorylates the N2B and PEVK (LeWinter & Granzier, 2013; Linke & Hamdani, 2014) segments of titin to reduce to effect conformational change and reduce stiffness (LeWinter, 2018). Reduced PKG activity drives cardiac hypertrophy (Kong & Blanton, 2013) through regulation of the calcineurin‐nuclear factor of activated T cells (NFAT) signaling axis. Nuclear factor of activated T cells target genes include pro‐hypertrophic mediators including brain natriuretic peptide (Fiedler et al., 2002). Reduced PKGI levels in cardiomyocytes and endothelial cells also lead to decreases in the anti‐inflammatory C‐type natriuretic peptide, which further sensitizes the myocardium to stress and inflammation (Moyes & Hobbs, 2019). Although the mechanistic link between nitrosative–oxidative stress and NLRP3 inflammasome activation has not yet been documented in HFpEF settings, it represents a worthwhile focus of future investigations. However, despite the mounting evidence from preclinical studies, the clinical investigations of broad antioxidants and anti‐nitrosative strategies have been largely negative (Borlaug et al., 2018; Mishra & Kass, 2021; Redfield et al., 2015).

### 4.3 Innate immune activation in HFpEF neutrophils

Increased blood pressure and oxidative stress further recruit inflammatory leukocytes including polymorphonuclear cells, notably neutrophils. Neutrophils, being the most abundant leukocyte in circulation, are the first to respond to injury. Neutrophils home to endothelial cells injured by systemic inflammation and secrete MPO to further exacerbate the inflammatory response. More so, MPO directly modulates endothelial cell inflammation by further limiting NO availability (Eiserich et al., 2002; Riehle & Bauersachs, 2019). Neutrophils, which account for more than half of all leukocytes are the first to mobilize to the injured area and release vesicles containing cytotoxic proteins like MPO (degranulation), generate reactive oxygen species (oxidative burst), phagocytose dead cells debris, and release extracellular traps (a process called NETosis). Neutrophils will also resolve inflammation by secreting neutrophil‐associated gelatinase lipocalin prime myeloid cells to more anti‐inflammatory phenotypes that may later contribute to TGFβ1‐mediated fibrosis (Horckmans et al., 2017; Hulsmans et al., 2018; Prabhu & Frangogiannis, 2016). Dying neutrophils potentiate the inflammatory response by shedding the IL‐6 receptor which activates nearby endothelial cells to amplify the inflammatory signals to recruit more leukocytes including Ly6C^hi^CCR2^hi^ monocytes and macrophages (Kratofil et al., 2017; Strassheim et al., 2019).

### 4.4 Macrophages

Macrophages are key mediators of homeostatic maintenance in cardiac tissue and play central roles in injury and repair. Under healthy conditions, the myocardium is populated with resident cardiac macrophages (rcMacs)‐a distinct macrophage population that arises from extramedullary hematopoiesis that takes up residence in the heart during fetal development (Honold & Nahrendorf, 2018). Under healthy conditions, rcMacs function to remove senescing or dying cells through phagocytosis, and mediate cardiac conduction through connexin 43 mediated coupling with cardiomyocytes (Hulsmans et al., 2017). Resident cardiac macrophages are largely anti‐inflammatory and can self‐renew. With age, rcMacs are less capable of clearing dead cell debris and self‐renewal. Resident cardiac macrophages senescence promotes replacement by peripheral macrophages which, with age also become more proinflammatory through secretion of IFNγ. Interleukin‐6 and TNFα (Oishi & Manabe, 2016) and less capable of antigen presentation (via downregulation of major histocompatibility complex II; MHC‐II). These dynamic changes are also seen in injury. Ischemic injury leads to major loss of rcMacs and an influx of peripheral monocytes. In HFpEF, macrophage numbers double compared to non‐HFpEF (DeBerge et al., 2019) most of which are peripheral‐derived. Right ventricular biopsy samples from HFpEF patients had a higher abundance of CD68^+^ infiltration compared to those from healthy individuals (Hahn et al., 2020).

While macrophages have classically been categorized as M1 and M2, maturing understanding points to a more complex reality. Indeed, macrophage phenotypes occupy a continuum of inflammatory and injury‐resolving capability. Analysis of blood from HFpEF patients identified a heterogeneity of monocyte subsets including acutely proinflammatory (mediated in part by NLRP3 signaling), cytotoxic phenotype to an anti‐inflammatory, and tissue‐remodeling capacities. The coexistence of proinflammatory and pro‐remodeling phenotypes in myocardial tissue promotes the feed‐forward cycle of inflammation and fibrosis. For instance, in HFpEF, significant recruitment of peripheral monocytes also occurs, however, resident macrophages also expand, in response to pressure overload, secrete higher levels of IL‐10, and promote diastolic dysfunction through profibrotic signaling including TGFβ/Smad signaling (Hulsmans et al., 2018). Profibrotic mediators include galectin‐3 (DeBerge et al., 2019; Suthahar et al., 2018), which correlate with worse clinical outcomes (Edelmann et al., 2015) and hospitalization. Similar findings implicating macrophages in HFpEF were further confirmed in a mouse model of hypertension, aldosterone‐infusion, and a renal failure‐induced mouse model of HFpEF (reviewed in DeBerge et al., 2019).

### 4.5 Adaptive immune activation in HFpEF

**FIGURE 4:**
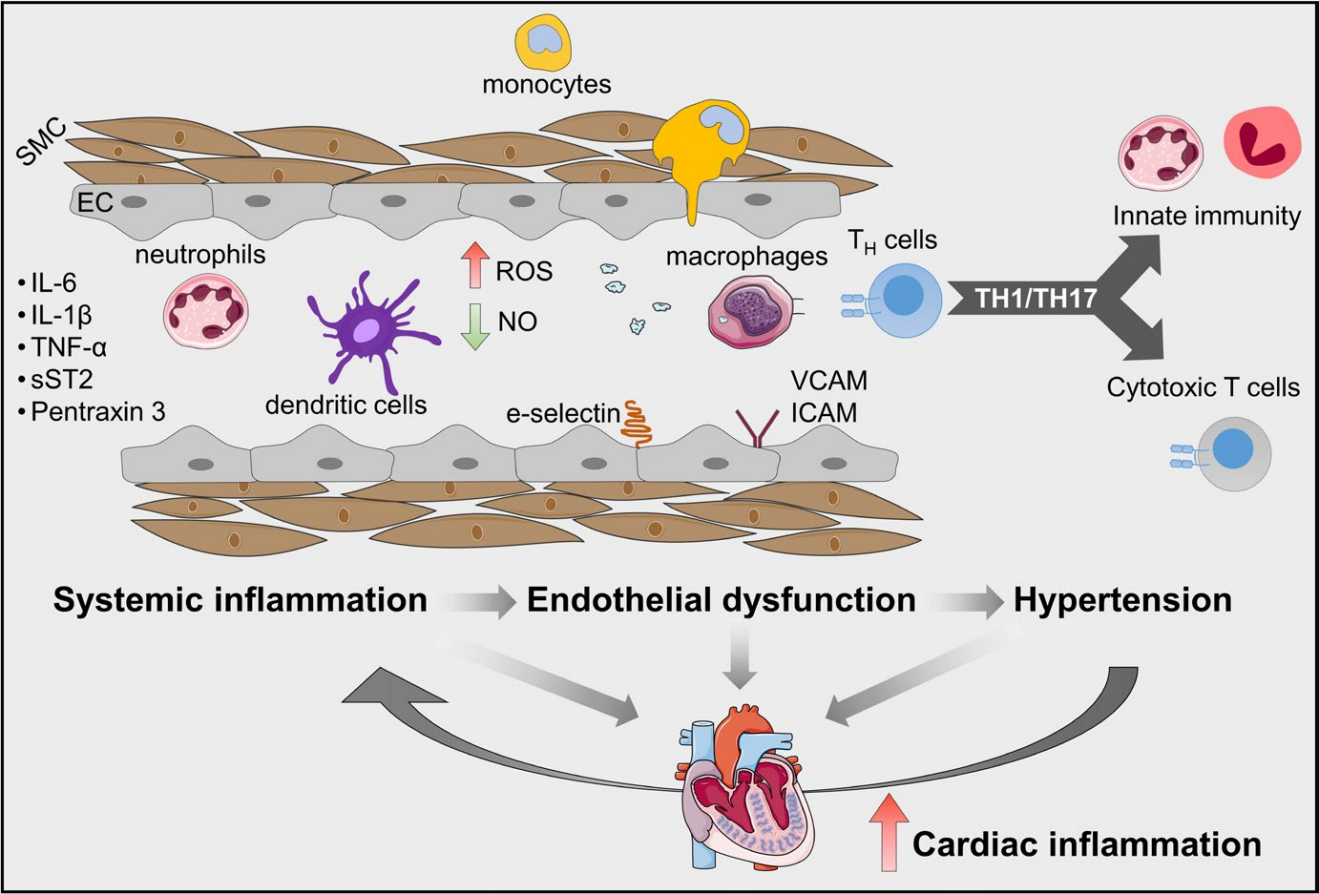
Impact of systemic and local inflammation on the pathogenesis of HFpEF. Multiple comorbidities induce a systemic inflammatory state leading to increased circulating levels of proinflammatory factors including interleukins‐6 and ‐1β (IL‐6 and IL‐1β), tumor necrosis factor (TNFα), soluble ST2 (sST2), and pentraxin 3 and upregulation of vascular cell adhesion molecule (VCAM), intercellular adhesion molecule (ICAM), and e‐selectin, which promote endothelial dysfunction. Subsequently, neutrophils and monocytes arrive and degranulate to amplify inflammation and oxidative stress. Macrophages arrive later and aid in clearing dead cell debris. As antigen‐presenting cells, they interface with helper T cells which, in the context of HFpEF polarize to TH1 and TH17 proinflammatory phenotypes. These helper T‐cell subsets orchestrate and perpetuate the inflammatory and fibrotic cycle. These events further amplify the dysfunction of endothelial and smooth muscle cells (EC and SMC, respectively), leading to increased production of reactive oxygen species (ROS) and decreased bioavailability of nitric oxide (NO), and consequently, hypertension. Systemic and coronary microvascular inflammation plays a decisive role in cardiac inflammation. A vicious inflammatory cycle is systemically and locally perpetuated to maintain high blood pressure and cardiac dysfunction. This figure was created in part with modified Servier Medical Art templates

Macrophages and other antigen‐presenting cells (APCs) serve as conduits between the innate and adaptive immune systems. Antigen‐presenting cells present antigens to CD4+ or helper T cells (T_H_) which orchestrate both the innate and adaptive immune response (Patel et al., 2018). The resultant polarity of T_H_ determines the ensemble of the immune response (Tokunaga et al., 2018). For instance, T_H_1, and T_H_17 (polarized by TNFα and IL17, respectively) have been implicated in HFpEF development (Blanton et al., 2019; Kallikourdis et al., 2017; Li et al., 2010). T cells traffic to the myocardium and are mobilized by endothelial selectins including ICAM1 and VCAM1 facilitate lymphocyte “rolling,” and ultimate anchoring to the endothelium and subsequent transendothelial migration into myocardial tissue. On the T‐cell side, chemokine receptors unique to T_H_ subsets bind selectins during homing including CXCR3 and CCR5 for T_H_1 and CCR6 for T_H_17, respectively. A third subset of T_H_ cells that regulate inflammatory responses (T regulatory cells; Treg) is reduced in HFpEF (Yue et al., 2019) which may drive further disease development (Kessler et al., 2020). Therefore, dysregulation of T_H_ subsets may play a significant role in HF pathology. Similar to the enrichment of heterogeneous populations of macrophages that occupy the myocardium, there are various subsets of proinflammatory T cells that occupy myocardial tissue and mediate inflammation and fibrosis. Thus, this complexity of innate and adaptive immune mediators may drive the complex microenvironment of the HFpEF (Blanton et al., 2019). Moreover, the efficacy of redirected T‐cell immunotherapy to specifically target cardiac fibrosis was recently demonstrated (Aghajanian et al., 2019), but this strategy has yet to be tested in HFpEF. Figure 4 summarizes systemic and local immune involvement in the pathogenesis of HFpEF.

## 5 THERAPEUTIC TARGETING OF NLRP3 INFLAMMASOMES

Preclinical studies motivated the development of new immunomodulatory therapies that target specific components of the immune response relevant to HF. For instance, therapeutic suppression of innate immune cells has been successfully achieved in ischemic and non‐ischemic cardiomyopathies by inhibition of the CCR2–CCL2 signaling axis. This was achieved using small‐molecule antagonists (Hilgendorf et al., 2014; Liao et al., 2018; Patel et al., 2018), monoclonal antibodies (Patel et al., 2018), and small interfering RNAs (Leuschner et al., 2011). Tailored therapies targeting the adaptive immune system, such as antibody‐based T cell (Bansal et al., 2017, 2019; Nevers et al., 2015) and B‐cell depletion (Cordero‐Reyes et al., 2016), have also yielded satisfactory outcomes. The safety and efficacy of immunomodulatory approaches have yet to be investigated in HFpEF clinical trials. A full discussion of the role of immunomodulation, autoimmunity, targeted anti‐cytokine therapies in HFrEF was reviewed elsewhere (Adamo et al., 2020; Jahns et al., 2010; Mann, 2015).

Despite advances in standard of care and pharmaceutical therapy, there are currently few effective therapies for HFpEF. Many of the emerging therapies in the pipeline target one of the many pathways involved in HFpEF pathologies such as activators of PKG (Borbély et al., 2009), beta‐receptor blockade (Kosmala et al., 2013), or inhibitors of late sodium current (Maier et al., 2013), and renin‐angiotensin‐aldosterone system (Edelmann et al., 2013). Thus far, these treatments have shown negative to modest clinical outcomes. The multisystemic disease of HFpEF syndrome (Shah et al., 2016) and heterogeneity HFpEF subphenotype (Shah et al., 2015) emphasizes the need for better‐targeted therapies for specific HFpEF subtypes. In light of the significant involvement of the myocardial inflammasome and the immune response and the paucity of immunomodulatory therapies, it is perhaps no surprise that current strategies remain unsuccessful.

Based on experimental and clinical evidence of the benefits of immunomodulatory therapy in HFpEF (Gallet et al., 2016; Glezeva & Baugh, 2014; Kessler et al., 2020), targeting NLRP3 inflammasome may represent a promising therapeutic approach. An ongoing clinical trial seeks to evaluate the expression of NLRP3 inflammasome components in the human HFpEF (NCT04269057). The overarching objective is to test whether blunting NLRP3 underlies the therapeutic benefits of angiotensin‐neprilysin inhibition in HFpEF (PARAGON‐HF, Solomon et al., 2019). Pharmacological strategies that specifically target the NLRP3 inflammasome has been investigated in several cardiovascular diseases. Indeed, a large number of compounds of NLRP3 inflammasome inhibitors have been reported and tested in proof‐of‐concept and clinical studies, including those that either directly inhibit NLRP3 or indirectly inhibit inflammasome components (Zahid et al., 2019). Specific targeting of NLRP3 by small molecules is cost‐effective and less invasive than direct cytokine blockade. Several such inhibitors have been developed to date (Zahid et al., 2019). Among the tested drugs in preclinical settings, previous studies showed that MCC950 and 16673‐34‐0 inhibited NLRP3 inflammasome activation with therapeutic properties in cardiac diseases (Carbone et al., 2018; van Hout et al., 2017).

To date, the diarylsulfonylurea compound MCC950 (Zahid et al., 2019) is considered the most potent and specific NLRP3 inhibitor, able to block both canonical and non‐canonical NLRP3 inflammasome activation. MCC950 also demonstrated therapeutic efficacy against atherosclerosis (van der Heijden et al., 2017), cardiac arrhythmias (Monnerat et al., 2016), myocardial infarction (van Hout et al., 2017), and diabetes (Zhai et al., 2018). However, despite the compelling efficacy of preclinical findings, including in large animal models (van Hout et al., 2017), a phase II clinical trial using MCC950 for rheumatoid arthritis was suspended due to hepatic toxicity (Mangan et al., 2018). Recently, OLT1177 (Dapansutrile) is a potent NLRP3 inhibitor (Toldo et al., 2019) with a more favorable safety profile, is currently being tested in clinical trials. Further studies and trials are needed to assess the efficacy, safety, and feasibility of this therapeutic approach.

Blockade of IL‐1 signaling has yielded successful clinical results and is currently being used in the treatment of NLRP3‐driven immunopathologies. For instance, canakinumab, an IL‐1β‐neutralizing antibody, and anakinra, a recombinant IL‐1 receptor antagonist is the top two most promising US Food and Drug Administration approved molecules (Dinarello et al., 2012) for the treatment of cardiovascular disorders. Canakinumab was tested in the CANTOS trial (Canakinumab Anti‐inflammatory Thrombosis Outcomes Study), which demonstrated that 150 mg canakinumab every 3 months significantly lower the recurrent rate of cardiovascular events in patients with atherosclerotic disease (Ridker et al., 2017). Interleukin‐1 blockade with anakinra has been also shown beneficial effects in patients with HF (Van Tassell et al., 2017) and reduced the systemic inflammatory response and improves aerobic exercise capacity in patients with HFpEF (D‐HART; Van Tassell et al., 2014). Subsequent phase II trial (D‐HART2) confirmed the reduction of C‐reactive protein and NT‐proBNP, and increased exercise capacity, but failed to improve peak oxygen consumption and ventilatory efficiency (Van Tassell et al., 2018). These latter findings are in contrast with the favorable outcomes found in the pilot study D‐HART, which might be due to the limited power of the phase I study and predominant obesity of participants in D‐HART2 that may have confounded exercise tolerance. Thus, a better understanding of the molecular pathophysiology of HFpEF phenotypes, including the inflammatory response, will be a key step toward enabling precision therapy for this heterogeneous syndrome.

### 5.1 Repurposed anti‐inflammatory therapies

Few therapies have been shown to alter HFpEF disease progression and prognosis. Thus, significant effort has already focused on drug repurposing. Ongoing trials include testing sodium‐glucose cotransporter 2 inhibitors (SGLT2i, empagliflozin, and dapagliflozin), a monoamine oxidase inhibitor (AZD4831), a xanthine oxidase inhibitor (allopurinol), and an inhibitor of uric acid transporter URAT1 (verinurad). Empagliflozin has shown profound cardioprotective benefits in HFrEF (Byrne et al., 2017) and HFpEF (Byrne et al., 2020). Sodium‐glucose cotransporter 2 inhibitors also decreases hospitalizations for HF and mortality in patients with type 2 diabetes at risk for atherosclerotic cardiovascular disease (Wiviott et al., 2019). Considering that a large portion of HFpEF individuals is obese, diabetic, and other metabolic diseases, targeting the metabolism‐induced inflammation offers a therapeutic opportunity for HFpEF patients. Interestingly, the beneficial cardiac effects of empagliflozin were associated with reduced cardiac inflammation via blunting activation of the NLRP3 inflammasome (Byrne et al., 2020). However, future investigations are needed to confirm its therapeutic efficacy. Table 1 lists the ongoing innervational trials of pharmacological therapies for HFpEF with immunomodulatory actions. Clinical trials testing other targets and device‐based approaches in HFpEF were recently reviewed (Mishra & Kass, 2021).

**TABLE 1:** Clinical trials of anti‐inflammatory drugs and repurposed agents for HFpEF

Study name	NCT identifier	Status	Agent
D‐HART2	NCT02173548	Completed	Anakinra
EMPEROR‐preserved	NCT03057951	Active	Empagliflozin
DETERMINE‐preserved	NCT03877224	Active	Dapagliflozin
DELIVER	NCT03619213	Recruiting	Dapagliflozin
PRESERVED‐HF	NCT03030235	Recruiting	Dapagliflozin
AMETHYST	NCT04327024	Recruiting	Allopurinol and Verinurad
MPO inhibitor A_Zeneca for HFpEF	NCT03611153	Recruiting	Myeloperoxidase inhibitor (AZD4831)
COLpEF	NCT04857931	Not yet recruiting	Colchicine

Inhibition of xanthine oxidase activity attenuates the generation of ROS and inflammasome activation in macrophages (Ives et al., 2015). Furthermore, MPO‐dependent oxidative stress is associated with elevated microvascular neutrophils in HFpEF patients (Hage et al., 2020). Despite the prominent roles of inflammasome signaling, its relevance to HFpEF in humans remains largely unexplored. Therefore, results from the ongoing clinical trials will reveal whether these repurposed agents are truly effective therapeutic options. Colchicine is a known anti‐inflammatory agent with clinical relevance for Gout and Behçet syndrome. Colchicine was recently shown to decrease cardiovascular‐related mortality in patients after myocardial infarction (Tardif et al., 2019). Thus, colchicine represents another potential repurposed therapy for HFpEF. Collectively, these futures studies may offer a paradigm shift in HFpEF management.

## 6 CONCLUSION

Chronic systemic inflammation is a key contributor (and potential trigger) to many age‐related disorders. Inflammasome activation and subsequent crosstalk with innate and adaptive immunity during aging is a prime signal to trigger multiple inflammatory signaling pathways that converge to impair heart function. The chronic and systemic inflammatory state is a central and prominent feature associated with HFpEF, which is disproportionately found in older individuals. Although there is evidence supporting “inflamed” hearts, this inflammatory HFpEF state is deeply influenced and perpetuated by non‐cardiac sources. The activation of NLRP3 inflammasome signaling in HFpEF remains circumstantial requiring future investigations. Hence, strategies aiming to inhibit cardiac proinflammatory pathways in HFpEF, including NLRP3 inflammasome signaling, may be appropriate therapeutic anti‐inflammatory interventions Thus, attenuation of inflammatory burden during aging may attenuate the severity of symptoms observed in HFpEF patients, which in turn can enhance the quality of life and life expectancy.

## CONFLICT OF INTEREST

None declared.

## AUTHOR CONTRIBUTIONS

TM, YNL, and AGI researched data for the article and drafted the manuscript. All authors have read and agreed to the published version of the manuscript.
